# Prospective acceptability of digital phenotyping among pregnant and parenting people with opioid use disorder: A multisite qualitative study

**DOI:** 10.3389/fpsyt.2023.1137071

**Published:** 2023-04-17

**Authors:** Elizabeth Charron, Ashley White, Kristi Carlston, Walitta Abdullah, Jacob D. Baylis, Stephanie Pierce, Michael S. Businelle, Adam J. Gordon, Elizabeth E. Krans, Marcela C. Smid, Gerald Cochran

**Affiliations:** ^1^Department of Health Promotion Sciences, Hudson College of Public Health, University of Oklahoma Health Sciences Center, Tulsa, OK, United States; ^2^Division of Epidemiology, Department of Internal Medicine, University of Utah School of Medicine, Salt Lake City, UT, United States; ^3^Department of Obstetrics, Gynecology and Reproductive Sciences, University of Pittsburgh, Pittsburgh, PA, United States; ^4^Section of Maternal-Fetal Medicine, Department of Obstetrics and Gynecology, College of Medicine, University of Oklahoma Health Sciences Center, Oklahoma City, OK, United States; ^5^TSET Health Promotion Research Center, Stephenson Cancer Center, University of Oklahoma Health Sciences Center, Oklahoma City, OK, United States; ^6^Informatics, Decision-Enhancement, and Analytic Sciences (IDEAS) Center, VA Salt Lake City Health Care System, Salt Lake City, UT, United States; ^7^Center for Perinatal Addiction Research, Education and Evidence-based Solutions (Magee CARES), Magee-Womens Research Institute, Pittsburgh, PA, United States; ^8^Division of Maternal Fetal Medicine, Department of Obstetrics and Gynecology, University of Utah Health, Salt Lake City, UT, United States

**Keywords:** acceptability, digital phenotyping, opioid use disorder, passive mobile sensing, pregnancy, postpartum period, qualitative

## Abstract

**Background:**

While medications for opioid use disorder (MOUD) effectively treat OUD during pregnancy and the postpartum period, poor treatment retention is common. Digital phenotyping, or passive sensing data captured from personal mobile devices, namely smartphones, provides an opportunity to understand behaviors, psychological states, and social influences contributing to perinatal MOUD non-retention. Given this novel area of investigation, we conducted a qualitative study to determine the acceptability of digital phenotyping among pregnant and parenting people with opioid use disorder (PPP-OUD).

**Methods:**

This study was guided by the Theoretical Framework of Acceptability (TFA). Within a clinical trial testing a behavioral health intervention for PPP-OUD, we used purposeful criterion sampling to recruit 11 participants who delivered a child in the past 12  months and received OUD treatment during pregnancy or the postpartum period. Data were collected through phone interviews using a structured interview guide based on four TFA constructs (affective attitude, burden, ethicality, self-efficacy). We used framework analysis to code, chart, and identify key patterns within the data.

**Results:**

Participants generally expressed positive attitudes about digital phenotyping and high self-efficacy and low anticipated burden to participate in studies that collect smartphone-based passive sensing data. Nonetheless, concerns were noted related to data privacy/security and sharing location information. Differences in participant assessments of burden were related to length of time required and level of remuneration to participate in a study. Interviewees voiced broad support for participating in a digital phenotyping study with known/trusted individuals but expressed concerns about third-party data sharing and government monitoring.

**Conclusion:**

Digital phenotyping methods were acceptable to PPP-OUD. Enhancements in acceptability include allowing participants to maintain control over which data are shared, limiting frequency of research contacts, aligning compensation with participant burden, and outlining data privacy/security protections on study materials.

## Introduction

In the past two decades, there have been substantial increases in the number of pregnancies affected by opioid use disorder (OUD) in the United States (US). From 1999 to 2017, the national prevalence of maternal OUD diagnoses at delivery increased from 1.5/1000 to 8.2/1000 deliveries (1, 2). Buprenorphine and methadone, collectively known as medications for OUD (MOUD), are evidence-based treatments for OUD, including during pregnancy and the postpartum period (3). However, about 50% of pregnant individuals who enter treatment do not receive MOUD pharmacotherapy (4–6). For those who do receive treatment, non-retention in treatment occurs at high rates in the postpartum period (7) and is potentially life-threatening. Between 50–90% of people experience opioid relapse within 1 month of discontinuing MOUD and this relapse is related to mortality (8).

Risk factors for MOUD treatment non-retention during pregnancy and the postpartum period include non-White race, younger age, alcohol and drug use/use disorders, missing healthcare appointments, and MOUD-related factors, such as lower duration or dose of treatment prior to delivery (9–12). Qualitative studies have identified additional retention barriers of parenting stress, mood disorders, discrimination for prenatal substance use, shame about having a substance-exposed infant, lack of autonomy over treatment decisions, and scrutiny from child welfare (13, 14). Despite this evidence, data used in these studies have limitations that prevent progress in the field. Patient-reported outcome and qualitative data may be influenced by self-reporting biases (15). Electronic health records and insurance claims are dependent on coding practices of sites or institutions, which often vary widely, and may contain incomplete, incorrect, or missing data (16, 17). Moreover, none of these data sources captures continuous, ecologically valid data in real time.

Digital phenotyping, defined as the “moment-by-moment quantification of the individual-level human phenotype *in situ* using data from personal digital devices,” is a novel approach for understanding social and behavioral dimensions of health and disease that overcomes many of the challenges inherent in traditional data collection methods (18). Digital phenotyping uses a smartphone’s embedded sensors to unobtrusively and longitudinally capture real time objective data on users as they interact with and move through their natural environments (19). This approach has been widely used in behavioral medicine and psychiatry to identify clinical markers of disease risk, monitor symptoms and outcomes, and tailor treatments in mental health and addictive disorders, including substance use overdose and relapse prediction, detection, and intervention (20, 21). Because data collection occurs in everyday contexts, it is an important tool for understanding behaviors of hard-to-reach populations who experience barriers to care and have intermittent healthcare utilization (22).

Digital phenotyping may provide new insights into the psychological states (stress, anxiety), behaviors (smoking, healthcare utilization), and social influences (interactions, isolation) contributing to MOUD non-retention among pregnant and parenting people with OUD (PPP-OUD), which if understood could offer treatment providers a window of opportunity to intervene before MOUD cessation occurs. Increasing treatment retention among PPP-OUD is critical to support long-term recovery, reduce risk of return to drug use and potential overdose, and provide a stable and safe home environment for parent and child. To our best knowledge, there is no prior or published research on the use of digital phenotyping with PPP-OUD. Given the nature of this type of data collection, understanding attitudes and perspectives of PPP-OUD about the digital phenotyping approach is an important step that needs to be completed before it can be used in a clinical research study. We conducted qualitative research using a theory-driven framework to assess the prospective, or anticipated, acceptability of digital phenotyping among PPP-OUD participating in randomized clinical trial. While smartphone-based digital phenotyping encompasses active (surveys) and passive (sensor) data collection (18), our inquiry was limited to the acceptability of passive sensor data collection (text/call logs, accelerometer, location/GPS).

## Materials and methods

### Theoretical framework

This work was informed and guided by the Theoretical Framework of Acceptability (TFA) (23), which Sekhon and colleagues developed to assess the degree to which people delivering or receiving an intervention consider it to be acceptable. The TFA consists of seven constructs (affective attitude, burden, ethicality, intervention coherence, opportunity costs, perceived effectiveness, and self-efficacy) that can be measured prospectively, concurrently, or retrospectively (23). We systematically applied four TFA constructs—affective attitude, burden, ethicality, and self-efficacy—that our team judged to be most relevant to measuring prospective acceptability of digital phenotyping (Table 1).

**Table 1:** Definitions of the Theoretical Framework of Acceptability (TFA) applied in this study.

TFA construct	Definition
Affective attitude	How an individual feels about digital phenotyping and participation in a study that collects passive sensor data
Burden	Perceived amount of effort required to participate in a study that collects passive sensor data
Ethicality	The extent to which digital phenotyping could be a good fit with the individual’s value system
Self-efficacy	The individual’s confidence that they can perform the behaviors needed to participate in a study that collects passive sensor data

### Study design and participants

The parent study of the current project was Optimizing Pregnancy and Treatment Interventions for Moms 2.0 (OPTI-Mom 2.0) (NCT03833245), a prospective clinical trial testing the efficacy of a patient navigator intervention to facilitate linkage to MOUD treatment engagement and psychosocial services among PPP-OUD (24). Details of the protocol can be viewed elsewhere (24) and are summarized here in brief to relate those most relevant to the current study. OPTI-Mom 2.0 participants included pregnant individuals ≤32 weeks of gestation presenting with OUD at University of Utah Hospital and Magee-Womens Hospital of UPMC, which are tertiary, academic medical centers providing general and specialty services to urban, suburban, and rural populations in Utah and Western Pennsylvania, respectively. Individuals who were taking MOUD >6 weeks, had documented manic or psychotic episode in the past 30 days, would not provide contact information, or planned to relocate after delivery were excluded (24). Data collection occurred between April 2019–January 2022 (24).

### Ethical approval

OPTI-Mom 2.0 obtained approval from the University of Utah Institutional Review Board (Protocol #00116398). This study’s participants were informed that the interviews were being conducted as part of the parent trial and that participation was voluntary. All provided consent prior to qualitative data collection.

### Sampling and recruitment

We conducted this qualitative study using purposeful criterion sampling, which involves identification of a sample based on pre-determined criteria of importance (25). Our criteria of interest were having a clinical diagnosis for OUD, delivering a newborn within the past 12 months, and receiving OUD treatment during pregnancy or the postpartum period. We selected participants who could best inform our research questions.

Two team members (KC and AW) supported recruitment by compiling a list of potential OPTI-Mom 2.0 study participants who met above criteria and invited them to participate in qualitative interviews through text message. Communications contained a description of the study and the first author’s (EC) contact information. Those who were interested to participate contacted EC through phone, text, or email. When possible, interviews were conducted immediately. No individuals refused participation. Recruitment and data collection were carried out simultaneously between March and May 2022.

### Data collection procedures

Data were collected by EC, a female, early career researcher with PhD-level training in qualitative inquiry and without any direct relation to research participants. Telephone interviews lasting approximately 30 min were conducted using a structured interview guide (Appendix A), which was developed by the research team and contained questions based on TFA constructs (23). Open-ended questions, such as “how do you feel about participating in a study that collects sensor data from your smartphone,” were used to elicit participant responses, with probing questions used to obtain more specific responses when needed. The investigative team piloted the interview guide prior to data collection with a certified physician assistant (PA-C) who works with PPP-OUD and subsequently revised the guide based on the PA-C’s feedback.

At the start of the interview, the interviewer informed participants of the study’s purpose and personal interest in the research topic. Field notes were taken during the interviews, and no other individuals were present besides the interviewer and participants. Participants received a $50 ClinCard payment as remuneration after interview completion. Upon observing informational redundancy during data collection, also known as data saturation (26, 27), we conducted one additional interview before ceasing recruitment and data collection to ensure that no new information came up. The final sample size included 11 participants, which is consistent with other research using criterion sampling methods with specialized populations of childbearing-aged people (28–30). Demographic and behavioral health data were abstracted from parent study participant information records.

### Data analysis and assurance of rigor

Interviews were audio recorded, transcribed verbatim, checked for accuracy against original recordings, and de-identified for analysis. Two team members (EC and AW) independently analyzed the qualitative transcripts by applying deductive framework analysis that included: (1) data familiarization; (2) identifying a thematic framework; (3) indexing; (4) charting; and (5) mapping and interpretation (31). First, we read interview transcripts many times to gain familiarity with the data. Next, we identified a framework, or categories, within which the data could be sorted and indexed. The categories were determined *a priori* based on TFA constructs from the interview guide. At the indexing stage, we linked the data to the thematic framework by applying deductive codes for TFA constructs. We also applied inductive codes for emergent themes. After coding three transcripts, EC and AW met to compare codes and discuss discrepancies in the application of the coding framework. Intercoder reliability was calculated for deductive codes with Cohen’s kappa, a measure of concordance that accounts for chance agreement (32), using Microsoft Excel version 16.68 (Microsoft Corp). Codes were considered concordant if both coders assigned the same TFA construct to the same segment of text. Because the kappa statistic for all deductive codes was 0.92, indicating an “almost perfect” level of agreement (33), both team members coded the remaining transcripts independently.

Next, we charted, or extracted, the data into a matrix format using a spreadsheet. At the final stage, we reviewed, reorganized, and collapsed the charted data to identify key patterns relative to the TFA constructs in Table 1. Descriptive statistics were calculated to describe the sample. Qualitative findings are organized by themes, including TFA constructs for deductive themes and emergent themes, and reported using participant quotes. Participant numbers (e.g., P1, P2) have been used in place of names to maintain anonymity. We used Microsoft Word version 16.65 (Microsoft Corp) and Microsoft Excel version 16.64 (Microsoft Corp) to organize, code, chart, and store the data. Reporting is in accordance with the COnsolidated criteria for REporting Qualitative research (*COREQ*) checklist (34).

## Results

### Sample characteristics

Among the 11 study participants, 9% were aged 18–25 years, 64% aged 25–34 years, and 27% aged 34 and older (Table 2). Most participants had public insurance (91%), were never married (45%), and had 1–2 previous children (55%). One participant (9%) identified as Black or African American race, one participant (9%) as Native Hawaiian or other Pacific Islander race, and nine participants (82%) as White race. A majority of participants reported tobacco (73%) and heroin use (91%). Over three-quarters (82%) of participants had depression.

**Table 2:** Characteristics of the sample.

Characteristics	*n* (%)
*Sociodemographic*	
Age, years	
18 to 25	1 (9%)
25 to 34	7 (64%)
34 and older	3 (27%)
Education	
Some high school	2 (18%)
High school diploma/equivalency	3 (27%)
Some college	4 (36%)
College graduate	2 (18%)
Insurance	
Public	10 (91%)
Private	1 (9%)
Marital status	
Married/coupled	4 (36%)
Widowed	2 (18%)
Never married	5 (45%)
Previous children	
0	2 (18%)
1–2	6 (55%)
≥3	3 (27%)
Ethnicity	
Hispanic or Latina	0 (0%)
Race	
White	9 (82%)
Black	1 (9%)
Native Hawaiian/Pacific Islander	1 (9%)
Study region	
Western Pennsylvania	3 (27%)
Utah	8 (73%)
Behavioral health	
Depression	9 (82%)
Anxiety	5 (45%)
Substance use	
Tobacco	8 (73%)
Alcohol	1 (9%)
Cannabis	5 (45%)
Heroin	10 (91%)
Methamphetamine	4 (36%)
Cocaine	1 (9%)

### Qualitative findings

#### Affective attitude

Participants largely expressed positive attitudes toward digital phenotyping and participating in a study that collects smartphone-based passive sensor data. As one participant (P1) explained:

“I don’t think there’s anything wrong with it. I think you have to have data for everything. Whether it’s smartphone data or getting it from a computer…no matter where the source comes from, you still have to have it in order to make things better and advanced and move forward.”

The main reasons for interviewees’ interest in participating in digital phenotyping research included receiving personal benefits, such as remuneration, and benefitting society. As one participant (P9) shared, *“I think the immediate is, what are they going to compensate me with, and then afterwards, if I might be actually helping other people.”*

Despite overall positive attitudes, participants reported concerns about privacy and security related to digital data collection and storage. P6 expressed concerns about “*somebody hacking in and collecting certain information.”* Other people reported apprehensions over data leakage, compromised personal information, receiving spam, and data being sold to third-party vendors or used for malicious purposes, such as identity theft. Interviewees also discussed their thoughts and opinions about which types sensor data collection would be acceptable. Content of text messages, phone calls, and photos were viewed as a *“total invasion of privacy”* (P4). Location/GPS data was acceptable to some but not to other individuals. As P10 commented, *“I do not know really about that…where I’ve been going and everything that people would know… I mean, it’s kind of invasive I guess.”*

Participants shared opinions about using their own phone vs. receiving a study phone for a digital phenotyping study. Those who preferred to use their own phone cited technical and personal familiarity as the main reasons for this choice. As P1 shared, *“I kind of like my own shit. I would not want to get adjusted to another phone.”* Those who favored a study phone provided numerous reasons for this preference, including having autonomy of the phone, not having a data plan to download apps, and having a second phone so that children could use their personal phone while the participant used a study phone. A few individuals did not have a preference and said that either option would be acceptable provided that they could keep the study phone at the end of the data collection period. As P11 explained, *“If I had to give it [study phone] back, then I’d rather just keep my phone than go through all the trouble, honestly.”* When probed about their preferred phone type, about half of participants preferred iPhone (n = 5) and the other half preferred Android (n = 6).

#### Burden

Interviewees reported low anticipated burden of participating in a digital phenotyping study. As P4 stated, *“For me personally, it would probably just like be in the back of my mind, and…it would just be kind of a silent thing going on, on my phone.”* They perceived no increased effort related to study participation during pregnancy, at the birth hospitalization, or at home after delivery, with the exception of P5 who expressed that study participation could be difficult during pregnancy due to morning sickness. Most people agreed that 12 months of participation after delivery would not be an excess burden. As P8 explained, “*You always have a little bit of time when their sleeping and stuff. I mean the phone is not a problem. Everyone has a phone all the time, even when they have a baby. So that will not be a problem.”*

Interviewees made recommendations to limit check-ins with the research staff and provide appropriate compensation relative to burden for study participants. There was no consensus about the frequency of check-ins. Biweekly or monthly was described as appropriate by many participants, whereas weekly was viewed as too frequent for some but not others. P4 shared, *“I feel like weekly would be almost too much unless it was like an email or a text message when I could answer it when I want it.”* Most participants preferred monetary remuneration. While a few desired to have their phone bill paid as a participation incentive, this preference was situation dependent. As P9 explained, *“I do not pay my cell phone bill because I’m on somebody else’s [plan]. So for me, personally, that [phone bill paid] would not be an advantage. I would rather get gas cards over that.”*

#### Ethicality

Participants described ethicality of digital phenotyping in different ways, including the appropriateness of smartphone-based sensor data collection in the context of a research study or more broadly within society. Whereas they expressed broad support for digital phenotyping research with known, trusted individuals, they expressed frustration about companies and government agencies accessing smartphone data without their consent. One participant (P2) explained:

“If it is not a study, it’s annoying to me and I don’t like it, because it causes a lot of things to pop up through your phone and phone calls and text messages. It’s very annoying. But for like a study purpose, I see it a little different because it’s specifically for one reason… I’ve been doing studies with them [the local university] for a while, and so I kind of feel comfortable with, you know, going in for a study, as opposed to how maybe the government collection watches you and stuff like that.”

Participants overwhelmingly agreed that smartphone apps harvesting data to improve ad targeting is unethical. They likewise expressed concerns about government tracking of cell phone locations. One participant (P4) commented:

“I don’t think it’s right that the government does that. No, I just, I don’t. I feel like that’s my privacy. And I mean, if you signed up for it, sure, because then you know about it. But I feel like a lot of the times people don’t know that they’re consenting to when they press accept on their phone.”

Interviewees conveyed the importance of knowing specific study details, such as types of data to be collected and how information would be secured and stored, before agreeing to study participation. As P10 explained, *“I would just have to, you know, hear exactly what kind of information they would be collecting, and everything like that, all the way down to the very bottom of it, you know what I mean.”*

#### Self-efficacy

Participants reported high self-efficacy to perform behaviors required to participate in a digital phenotyping study. None expressed concerns about keeping smartphones with them, charged, and turned on at all times. As P3 stated, *“Everybody has their phone on them all the time.”* P9, however, commented that keeping a phone charged may be challenging for individuals who are homeless: *“They probably would not be able to…keep the battery life because they are charging their phone at McDonald’s* versus *in their home or in their car.”* Despite participants’ confidence, over the course of the interviews they described instances of losing or breaking a phone, allowing children to use the phone, not having a data plan to download an app, and not having autonomy of the phone. As P3 shared, *“I’m going through a divorce right now… My ex, like, took my kids. I have not seen them for 4 months, and then yesterday…he turned my phone off.”* Another participant (P6) explained:

“My kids tend to use the phone, like when I’m napping, or when we’re at a doctor’s appointment. I will give the phone to them so that they’re distracted, or like at church when I’m trying to keep them down. But other than that, I have my phone.”

#### Potential interventions

Participants perceived that digital phenotyping was a good opportunity to learn about their and others’ behaviors. A participant (P7) commented, *“I think we are going to learn a lot from it. We’re going to learn a lot of people’s behaviors, a lot of their habits, a lot of their freaking out.”* Though not specifically asked, interviewees suggested how smartphone-based sensor data could be used to benefit study participants. Making data available for participants to track and improve their personal health behaviors, including sleeping patterns, screen time, and social media activity, was frequently mentioned. P2 suggested, *“If you all would show me, like, this is how much you slept, then I would know, and I could maybe make some changes in my lifestyle.”* One participant (P6) also proposed a mobile text messaging alert to remind participants to take their MOUD treatment: *“If I had a little notification or you guys pinged me and were like, oh, it’s past your medication time… Then I would be like okay, you know, like a quick little reminder—then I would not forget it.”*

## Discussion

This study applied the TFA to assess the acceptability of using digital phenotyping methods, specifically data collected passively from smartphone sensors, with PPP-OUD. We found that digital phenotyping approaches are generally acceptable to PPP-OUD when individuals accessing the data are known and trusted and participants maintain control over which data are shared.

Participants’ attitudes and perspectives about digital phenotyping were influenced by the perceived universality of smartphones, their high self-efficacy for using smartphone technology and apps, and the type of sensor data to be collected. Approximately half of participants perceived that sharing location/GPS data was too intrusive. This is in line with findings from past research conducted among patients in drug treatment that reported 46 and 62% of participants were not comfortable with smartphone apps gathering location/GPS data (35, 36).

In 2021, 85% of American adults owned a smartphone (37), and rates of smartphone ownership are similarly high among people with substance use disorders (SUDs) (36). One recent US study of outpatient methadone clinic patients found that 93.1% of people owned a smartphone and 86.2% currently used apps (36). While 100% of this study’s participants used smartphones, several reported not having autonomy over their device or data plan. This is an important consideration in that it could negatively impact data quality. Future digital phenotyping studies with PPP-OUD may overcome this issue by offering participants the option to use a study phone.

The anticipated burden of participating in a digital phenotyping study during pregnancy and the postpartum period was low among study participants. Most people indicated that 12 months of participation after delivery was acceptable provided that remuneration for participation over time was appropriate and research contacts were minimized. While financial incentives were the favored method of remuneration, alternative compensation strategies, such as paying cell phone bills or allowing participants to keep the study phone, were popular and could be used in future studies to encourage participation from a wider pool of people. Limiting research contacts may also increase retention as some research suggests that contact frequency is associated with attrition in longitudinal studies (38).

Participants believed that digital phenotyping was ethical from a research perspective, but they expressed concerns about data security and privacy, such as how data would be used, stored, and protected from being downloaded by or shared with outside entities. Notably, participants’ concerns were mitigated by having institutional trust and perceptions that research team members would ensure privacy and confidentially of their data. There is strong agreement among experts of various disciplines that privacy, security, and trust are central ethical issues in digital phenotyping (39). These issues may be particularly salient for PPP with SUDs because they are highly vulnerable to stigma, discrimination, criminalization, and child welfare system involvement (40). PPP-OUD participating in digital phenotyping research should be reassured that data will not be used to test for or report prenatal drug use to child protective services or law enforcement, which could be discussed alongside procedures on data usage, storage, and security during the consent process. Moreover, all studies of this nature should apply for a Certificate of Confidentiality that prevents government agencies (e.g., police) from obtaining study data.

Our findings also provide initial insight into potential interventions that may be used to decrease perinatal MOUD non-retention. One participant perceived that text messaging would be valuable for facilitating MOUD adherence. Text messaging interventions can be simple and cost-effective in the promotion of medication adherence and improve health outcomes among people with SUDs and mental health disorders (41). One study showed that text messaging reminders were acceptable to and improved appointment adherence for 95% of patients in an outpatient buprenorphine program (42). Given that PPP-OUD often have psychosocial comorbidities and experience structural barriers, such as addiction stigma, poverty, and lack of housing, that can impact treatment retention (43), text messaging reminders alone may not provide substantial and prolonged benefits. Thus, simple smartphone-based interventions (e.g., text messaging) should be combined with previously tested intervention components, such as medication self-monitoring and psychosocial support. Multilevel interventions that target interrelated behavior, social, and structural factors impeding MOUD retention are also needed.

### Strengths and limitations

A primary strength of this study includes the use of a guiding theoretical framework to evaluate acceptability. As with all qualitative research, the main limitation of this study is generalizability. We used purposeful criterion sampling to select a narrow sample of PPP-OUD. While the sampling method was appropriate and improved the rigor of this research, we cannot extrapolate findings to PPP-OUD outside this sample. In addition, all participants in this study were of non-Hispanic ethnicity, and most of White race, and responses given by these individuals may not generalize to persons of other ethnic and racial groups. Another limitation is that many participants were still engaged with parent study and motivated to speak with the interviewer. Views of PPP-OUD who were lost to follow-up in the parent study, not interested to discuss digital phenotyping, or not eligible for the parent study may have had dissimilar perspectives and attitudes. Moreover, we did not conduct member checking with participants after transcribing the interviews in order to establish credibility of findings; however, this decision was made *a priori* due to time and resource constraints. Finally, we coded qualitative data according to *a priori* codes based on TFA constructs, which have a considerable amount of overlap. Other investigators may have applied codes and constructed themes in an alternate manner.

## Conclusion

Digital phenotyping is a novel approach for understanding factors contributing to MOUD non-retention, however information has been lacking on whether it is suitable to PPP-OUD. This study provides initial evidence that digital phenotyping methods may be acceptable to PPP-OUD when people accessing the data are known and trusted and participants maintain control over which data are shared. Enhancements to acceptability include allowing participants to maintain control over which data are shared, limiting frequency of research contacts, aligning compensation with participant burden, and outlining data privacy/security protections on study materials.

## Data availability statement

The datasets presented in this article are not readily available because due to the nature of this research participants of this study were not asked and did not agree for their data to be shared publicly. Requests to access the datasets should be directed to elizabeth-charron@ouhsc.edu.

## Ethics statement

The studies involving human participants were reviewed and approved by University of Utah Institutional Review Board. The patients/participants provided their written informed consent to participate in this study.

## Author contributions

EC conceptualized the study, developed the methodology, conducted the investigation, and prepared the first draft of this manuscript. EC, AW, and KC recruited participants. EC and AW analyzed the data. EC, AW, KC, WA, JB, SP, MB, AG, EK, MS, and GC critically reviewed and revised the manuscript for important intellectual content. All authors accept responsibility for the integrity of the work reported and approved the final version of this manuscript.

## Funding

This study was supported by a grant from the Centers for Disease Control and Prevention (R01CE002996; PI Cochran). Cochran and Gordon are supported by a grant from the National Institute on Drug Abuse (1UG1DA049444–01 PI: Gordon/Cochran). The content is solely the responsibility of the authors and does not necessarily represent the official views of the funder.

## Conflict of interest

The authors declare that the research was conducted in the absence of any commercial or financial relationships that could be construed as a potential conflict of interest.

## Publisher’s note

All claims expressed in this article are solely those of the authors and do not necessarily represent those of their affiliated organizations, or those of the publisher, the editors and the reviewers. Any product that may be evaluated in this article, or claim that may be made by its manufacturer, is not guaranteed or endorsed by the publisher.
