# Supplementary material for: Prospective acceptability of digital phenotyping among pregnant and parenting people with opioid use disorder: A multisite qualitative study
Source: Front Psychiatry. 2023 Apr 17;14:1137071. doi: 10.3389/fpsyt.2023.1137071 (PMC10149825; doi:10.3389/fpsyt.2023.1137071)
Supplement: Supplementary file 1 [file Data_Sheet_1.pdf]

Supplementary Material

Prospective acceptability of digital phenotyping among pregnant and parenting people with opioid use disorder: A multisite qualitative study

Authors: Elizabeth Charron, MPH, PhD[1,2*], Ashley White, MA, CMHC[2], Kristi Carlston[2], Walitta Abdullah[3], Jacob D. Baylis, MPH[2], Stephanie Pierce, MD, MS[4], Michael S. Businelle, PhD[5], Adam J. Gordon, MD, MPH [2,6], Elizabeth E. Krans, MD, MSc[3,7], Marcela C. Smid, MD, MS, MA[2,8], Gerald Cochran, MSW, PhD[2]

[1] Department of Health Promotion Sciences, Hudson College of Public Health, University of Oklahoma Health Sciences Center, Tulsa, OK, United States

[2] Division of Epidemiology, Department of Internal Medicine, University of Utah School of Medicine, Salt Lake City, UT, United States

[3] Department of Obstetrics, Gynecology and Reproductive Sciences, University of Pittsburgh, Pittsburgh, PA, United States

[4] Section of Maternal-Fetal Medicine, Department of Obstetrics and Gynecology, College of Medicine, University of Oklahoma Health Sciences Center, Oklahoma City, OK, United States

[5] TSET Health Promotion Research Center, Stephenson Cancer Center, University of Oklahoma Health Sciences Center, Oklahoma City, OK, United States

[6] Informatics, Decision-Enhancement, and Analytic Sciences (IDEAS) Center, VA Salt Lake City Health Care System, Salt Lake City, UT, United States

[7] Center for Perinatal Addiction Research, Education and Evidence-based Solutions (Magee CARES), Magee-Womens Research Institute, Pittsburgh, PA, United States

[8] Division of Maternal Fetal Medicine, Department of Obstetrics and Gynecology, University of Utah Health, Salt Lake City, UT, United States [h]

*** Correspondence:**
Elizabeth (Betsy) Charron
elizabeth-charron@ouhsc.edu

Structured Interview Guide

TFA Construct	Main Question	Probes
Affective Attitude	How would you feel about participating in a study that captured passive data from your smartphone? Passive data could include how many minutes spent talking, how many text messages sent, locations that you traveled, and amount of screen time. Data would not include the specific content of your phone calls, text messages, or photos.	- Type of data<br>- Advantages/disadvantages<br>- Personal phone vs study phone
Burden	How burdensome would it be to participate in a study that captured passive data from your smartphone?	- Length of data collection<br>- Timepoint of data collection<br>- Ease burden (incentives, time)
Ethicality	What are your beliefs about smartphone data collection in general?	- Appropriateness<br>- Fit with values<br>- Concerns or worries (privacy, security)
Self-efficacy	How confident would you be to participate in a study that captured passive data from your smartphone?	- Anticipated issues (keeping phone charged, lose phone, someone else using phone)

Abbreviations: TFA, Theoretical Framework of Acceptability

COREQ (COnsolidated criteria for REporting Qualitative research) Checklist

A checklist of items that should be included in reports of qualitative research. You must report the page number in your manuscript where you consider each of the items listed in this checklist. If you have not included this information, either revise your manuscript accordingly before submitting or note N/A.

Topic	Item No.	Guide Questions/Description	Reported on Page No.
**Domain 1: Research team and reflexivity**			
Personal characteristics			
Interviewer/facilitator	1	Which author/s conducted the interview or focus group?	3
Credentials	2	What were the researcher's credentials? E.g. PhD, MD	3
Occupation	3	What was their occupation at the time of the study?	3
Gender	4	Was the researcher male or female?	3
Experience and training	5	What experience or training did the researcher have?	3
Relationship with participants			
Relationship established	6	Was a relationship established prior to study commencement?	3
Participant knowledge of the interviewer	7	What did the participants know about the researcher? e.g. personal goals, reasons for doing the research	3
Interviewer characteristics	8	What characteristics were reported about the inter viewer/facilitator? e.g. Bias, assumptions, reasons and interests in the research topic	3
**Domain 2: Study design**			
Theoretical framework			
Methodological orientation and Theory	9	What methodological orientation was stated to underpin the study? e.g. grounded theory, discourse analysis, ethnography, phenomenology, content analysis	2
Participant selection			
Sampling	10	How were participants selected? e.g. purposive, convenience, consecutive, snowball	3
Method of approach	11	How were participants approached? e.g. face-to-face, telephone, mail, email	3
Sample size	12	How many participants were in the study?	3
Non-participation	13	How many people refused to participate or dropped out? Reasons?	3
Setting			
Setting of data collection	14	Where was the data collected? e.g. home, clinic, workplace	3
Presence of non-participants	15	Was anyone else present besides the participants and researchers?	3
Description of sample	16	What are the important characteristics of the sample? e.g. demographic data, date	4
Data collection			
Interview guide	17	Were questions, prompts, guides provided by the authors? Was it pilot tested?	3
Repeat interviews	18	Were repeat inter views carried out? If yes, how many?	N/A
Audio/visual recording	19	Did the research use audio or visual recording to collect the data?	3
Field notes	20	Were field notes made during and/or after the inter view or focus group?	3
Duration	21	What was the duration of the inter views or focus group?	3
Data saturation	22	Was data saturation discussed?	3
Transcripts returned	23	Were transcripts returned to participants for comment and/or	6

Topic	Item No.	Guide Questions/Description	Reported on Page No.
		correction?	
**Domain 3: analysis and findings**			
Data analysis			
Number of data coders	24	How many data coders coded the data?	3
Description of the coding tree	25	Did authors provide a description of the coding tree?	3-4
Derivation of themes	26	Were themes identified in advance or derived from the data?	3-4
Software	27	What software, if applicable, was used to manage the data?	4
Participant checking	28	Did participants provide feedback on the findings?	6
Reporting			
Quotations presented	29	Were participant quotations presented to illustrate the themes/findings? Was each quotation identified? e.g. participant number	4-6
Data and findings consistent	30	Was there consistency between the data presented and the findings?	4-7
Clarity of major themes	31	Were major themes clearly presented in the findings?	4-6
Clarity of minor themes	32	Is there a description of diverse cases or discussion of minor themes?	4-6

Developed from: Tong A, Sainsbury P, Craig J. Consolidated criteria for reporting qualitative research (COREQ): a 32-item checklist for interviews and focus groups. *International Journal for Quality in Health Care*. 2007. Volume 19, Number 6: pp. 349 – 357

Once you have completed this checklist, please save a copy and upload it as part of your submission. DO NOT include this checklist as part of the main manuscript document. It must be uploaded as a separate file.
